# Circulating miRNA Expression Profiling in Primary Aldosteronism

**DOI:** 10.3389/fendo.2019.00739

**Published:** 2019-10-29

**Authors:** Abel Decmann, Gábor Nyírö, Ottó Darvasi, Péter Turai, Irina Bancos, Ravinder Jeet Kaur, Raffaele Pezzani, Maurizio Iacobone, Ivana Kraljevic, Darko Kastelan, Mirko Parasiliti-Caprino, Mauro Maccario, Nina Nirschl, Daniel Heinrich, Martin Reincke, Attila Patócs, Peter Igaz

**Affiliations:** ^1^2nd Department of Internal Medicine, Faculty of Medicine, Semmelweis University, Budapest, Hungary; ^2^MTA-SE Molecular Medicine Research Group, Hungarian Academy of Sciences and Semmelweis University, Budapest, Hungary; ^3^Hereditary Endocrine Tumors Research Group, Hungarian Academy of Sciences and Semmelweis University, Budapest, Hungary; ^4^Division of Endocrinology, Diabetes, Metabolism and Nutrition, Department of Internal Medicine, Mayo Clinic, Rochester, MN, United States; ^5^Endocrinology Unit, Department of Medicine, University of Padua, Padova, Italy; ^6^Minimally Invasive Endocrine Surgery Unit, Department of Surgery, Oncology and Gastroenterology (DISCOG), University of Padova, Padova, Italy; ^7^Department of Endocrinology, University Hospital Centre Zagreb, Zagreb, Croatia; ^8^Division of Endocrinology, Diabetology and Metabolism, Department of Medical Sciences, University of Turin, Turin, Italy; ^9^Medizinische Klinik und Poliklinik IV, Ludwig Maximilian University Munich, Munich, Germany

**Keywords:** adrenal, primary aldosteronism, microRNA, biomarker, aldosterone-producing adenoma, bilateral adrenal hyperplasia

## Abstract

**Objective:** Primary aldosteronism is a major cause of secondary hypertension. Its two principal forms are bilateral adrenal hyperplasia (BAH) and aldosterone-producing adenoma (APA) whose differentiation is clinically pivotal. There is a major clinical need for a reliable and easily accessible diagnostic biomarker for case identification and subtyping. Circulating microRNAs were shown to be useful as minimally invasive diagnostic markers. Our aim was to determine and compare the circulating microRNA expression profiles of adenoma and hyperplasia plasma samples, and to evaluate their applicability as minimally invasive markers.

**Methods:** One hundred and twenty-three samples from primary aldosteronism patients were included. Next-generation sequencing was performed on 30 EDTA-anticoagulated plasma samples (discovery cohort). Significantly differently expressed miRNAs were validated by real-time reverse transcription-qPCR in an independent validation cohort (93 samples).

**Results:** We have found relative overexpression of *miR-30e-5p, miR-30d-5p, miR-223-3p*, and *miR-7-5p* in hyperplasia compared to adenoma by next-generation sequencing. Validation by qRT-PCR confirmed significant overexpression of *hsa-miR-30e-5p, hsa-miR-30d-5p*, and *hsa-miR-7-5p* in hyperplasia samples. Regarding the microRNA expressional variations, adenoma is more heterogeneous at the miRNA level compared to hyperplasia.

**Conclusion:** Three microRNAs were significantly overexpressed in hyperplasia samples compared to adenoma samples, but their sensitivity and specificity values are not good enough for introduction to clinical practice.

## Introduction

Primary aldosteronism (PA) is a major cause of secondary hypertension affecting 5–13% of hypertensive patients (1–3). The two principal forms of PA are bilateral adrenal hyperplasia (BAH) and aldosterone-producing adenoma (APA) accounting for 60–70 and 30–40% of all PA cases, respectively (4). Somatic mutations of *KCNJ5* (Potassium Voltage-Gated Channel Subfamily J Member 5) (Gly151Arg or Leu168ARg) gene are present in 34–43% of APAs (5–9). Mutations in *ATP1A1* (ATPase Na^+^/K^+^ transporting Subunit Alpha 1; in 5.3–17%), *ATP2B3* (ATPase Plasma Membrane Ca^++^ Transporting 3; in 1.7–4%), *CACNA1D* (Calcium Voltage-Gated Channel Subunit Alpha 1D; in 9.3–21%), and *CTNNB1* (Catenin Beta 1; in 2.1–5.1%) genes are responsible for a smaller portion of APAs (10–14). Nanba et al. found somatic mutations in aldosterone-driving genes in 88% of APAs by comprehensive NGS of CYP11B2 (aldosterone synthase)-expressing adrenal tumors (13). Moreover, tumors harboring *CACNA1D* mutation were found to be smaller than tumors with *KCNJ5* mutations (14). In contrast, the pathogenesis of BAH is largely unknown that is mostly related to the lack of tissue samples for analysis since it is mostly left unoperated.

Due to the difference in treatment strategies (surgical resection for APA, mineralocorticoid antagonists for BAH), differentiation of APA and BAH is of pivotal clinical relevance. Adrenal venous sampling (AVS) is considered to be the gold standard for the differentiation of the two clinical entities, but it is invasive, requires great expertise and unfortunately unavailable in many centers (15–17). The recent SPARTACUS trial has challenged the superiority of AVS over imaging, but its findings are debated (18). The need for a reliable and easily accessible diagnostic biomarker enabling their differentiation is critical to assure the best clinical management for patients with primary hyperaldosteronism.

MicroRNAs (miRNA, miR) in their mature forms are short (19–25 nucleotide long), single-stranded, non-coding RNA molecules involved in the gene expression mostly at the post-transcriptional level. MiRNAs are expressed in a tissue-specific manner (19), and are also secreted in various body fluids; as such, miRNAs hold promise as potential diagnostic biomarkers, as a component of liquid biopsy (20, 21). Our aim has been to perform profiling of circulating plasma miRNAs in AVS-confirmed samples of patients with primary hyperaldosteronism in order to determine biomarkers for differentiation of APA vs. BAH.

The aim of our study was to determine and compare the circulating microRNA expression profiles of APA and hyperplasia plasma samples, and to evaluate their applicability as minimally invasive markers in replacing AVS in the diagnostics of PA.

## Materials and Methods

### Sample Collection and Ethics Approval

A total of 123 EDTA-anticoagulated plasma samples were used (Table 1). Altogether, 61 APA and 62 BAH samples were included in the study. Seventy-two male and Fifty-one female patients' samples were included. The average age has been 54.17 for women and 49.39 for men. The sex of patients was not considered as a factor in the statistical analysis of the data. Diagnosis of PA was established according to current guidelines (22). APA and BAH were differentiated by AVS with or without ACTH stimulation (Table 2). Lateralization index (LI) was used to differentiate between the two entities [(left side cortisol/left side aldosterone)/(right side cortisol/right side aldosterone)]. If LI was between 0.33 and 3, the sample was considered as BAH, while if it was more than 4 or <0.25, the sample was considered to be APA. Samples from APA patients were collected preoperatively. Genetic results of APA samples were available only for a minority of cases. Samples with lateralization index of 0.25–0.33 and 3–4 were considered to be in the zones of overlap, thus not included in the study (23). The study was approved by the Ethical Committee of the Hungarian Health Council. All experiments were performed according to relevant guidelines and protocols, and from all the involved patients written informed consent was obtained.

**Table 1:** Patient data of the 123 samples included.

**Sample**	**Diagnosis**	**Cohort**	**Age at diagnosis (range)**	**Tumor size (mm), laterality**	**Aldosterone AVS right nmol/L**	**Aldosterone AVS left nmol/L**	**Cortisol AVS right nmol/L**	**Cortisol AVS left nmol/L**	**Lateralization index**	**Date of sampling**
1	BAH	NGS	40–45	15, right	22.27	43.63	62.39	127.6	1.04	2018
2	BAH	NGS	60–65	Normal	16.68	12.69	177.55	121.33	0.9	2018
3	BAH	NGS	30–35	12, left	24.52	9.47	67.61	22.8	0.87	2018
4	BAH	NGS	56–60	10, right	14.9	9.35	75.55	52.13	1.1	2018
5	BAH	NGS	36–40	Normal	15.31	12.17	175.13	99.11	0.71	2018
6	BAH	NGS	36–40	Normal	55.12	54.66	126.37	115.2	0.92	2018
7	BAH	NGS	40–45	Normal	26.42	20.8	339.67	157.47	0.59	2018
8	BAH	NGS	56–60	Normal	23.37	14.92	160.04	84.86	0.83	2018
9	BAH	NGS	65–70	Normal	7.82	7.13	99.8	83.96	0.92	2018
10	BAH	NGS	46–50	10/19, right/left	103.76	42.45	326.61	225.48	1.69	2017
11	BAH	NGS	60–65	9/7, right/left	44.39	47.16	176.54	144.64	0.77	2016
12	BAH	NGS	70–75	5/15, right/left	835.06	882.23	387.88	249.76	0.61	2015
13	BAH	NGS	66–70	5/5, right/left	174.78	66.58	256.29	92.08	0.94	2015
14	BAH	NGS	50–55	16, left	9.61	6.39	221.49	167.48	1.14	2016
15	APA	NGS	70–75	Normal	1.36	24.33	35.53	17.4	0.03	2018
16	APA	NGS	86–90	15, right	31.34	1.32	6.89	6.53	22.58	2013
17	APA	NGS	40–45	12, left	0.39	38.9	245.05	276.23	0.01	2013
18	APA	NGS	56–60	12, left	57.24	5.28	85.19	193.94	24.68	2014
19	APA	NGS	46–50	15, right	20.3	4.4	108.03	160.23	6.85	2014
20	APA	NGS	40–45	10, left	1.25	225	6.89	12.33	0.01	2014
21	APA	NGS	50–55	Normal	5.09	236	469.8	482.85	0.02	2016
22	APA	NGS	56–60	8, right	1170.76	9.93	327.7	248.68	89.45	2017
23	APA	NGS	30–35	14, right	108.2	2	97.15	27.55	15.36	2017
24	APA	NGS	56–60	29, right	78.51	2.64	271.51	81.56	8.95	2015
25	APA	NGS	46–50	5/5, right/left	33.29	169.23	416.88	160.23	0.08	2017
26	APA	NGS	40–45	10, right	1942.01	6.94	369.75	179.08	135.61	2017
27	APA	NGS	50–55	5/10, right/left	0.39	310.72	5.44	207.71	0.05	2016
28	APA	NGS	46–50	7/9, right/left	61.03	4.72	315.01	122.16	5.02	2015
29	APA	NGS	56–60	6/7, right/left	55.49	124.84	213.51	88.45	0.18	2016
30	APA	NGS	60–65	12, left	30.52	63.81	219.31	96.43	0.21	2017
31	BAH	Validation	40–45	Normal	41.07	13.69	539.04	214.6	1.19	2018
32	BAH	Validation	30–35	Normal	48.49	21	390.78	181.98	1.08	2018
33	BAH	Validation	40–45	Normal	37.79	13.6	192.13	101.86	1.47	2018
34	BAH	Validation	70–75	26, left	65.99	17.58	553.9	193.21	1.31	2018
35	BAH	Validation	60–65	23, left	5.05	38.09	22.48	170.01	1	2018
36	BAH	Validation	46–50	Normal	33.69	39.79	328.79	161.31	0.42	2018
37	BAH	Validation	35–40	Normal	50.19	41.29	234.9	312.84	1.62	2018
38	BAH	Validation	60–65	2, left	18.3	14.55	174	104.76	0.76	2018
39	BAH	Validation	50–55	20, right	6.49	3.9	94.25	106.58	1.88	2014
40	BAH	Validation	36–40	20, right	0.33	1.08	4.21	7.21	0.52	2015
41	BAH	Validation	66–70	7, left	3.28	5.68	48.58	36.61	0.44	2018
42	BAH	Validation	60–65	Normal	0.5	17.37	15.23	199.01	0.38	2014
43	BAH	Validation	46–50	Normal	1.04	3.38	19.21	56.91	0.91	2014
44	BAH	Validation	56–60	Normal	66.03	5.58	537.23	55.46	1.22	2014
45	BAH	Validation	50–55	15, left	4.77	13.07	20.3	89.18	1.6	2015
46	BAH	Validation	40–45	10, left	0.69	22.86	17.04	711.96	1.26	2015
47	BAH	Validation	56–60	Normal	1.44	1.56	21.39	20.3	0.87	2015
48	BAH	Validation	46–50	Normal	3.72	107.2	67.06	1255.71	0.65	2016
49	BAH	Validation	50–55	Normal	17.01	30.93	176.9	344.74	1.07	2016
50	BAH	Validation	56–60	Normal	2.63	3.97	6.89	11.24	1.08	2015
51	BAH	Validation	40–45	Normal	9.73	7.74	10.88	17.04	1.97	2013
52	BAH	Validation	40–45	Normal	2.8	4.26	40.24	95.34	1.56	2013
53	BAH	Validation	36–40	Normal	6.18	1.04	233.09	27.91	0.71	2012
54	BAH	Validation	56–60	7, right	4.43	12.13	117.09	208.8	0.65	2012
55	BAH	Validation	50–55	Normal	4.82	15.58	199.01	362.5	0.56	2011
56	BAH	Validation	30–35	Normal	6.46	1.14	83.05	19.68	1.35	2011
57	BAH	Validation	40–45	Left 1.3 cm	1.62	36.62	40.6	347.28	0.38	2011
58	BAH	Validation	60–65	Normal	15.05	9.95	47.96	61.66	1.94	2010
59	BAH	Validation	40–45	10, left	5.27	53.82	11.64	242.19	2.04	2010
60	BAH	Validation	45–50	Normal	3.68	2.44	206.01	244.47	1.79	2010
61	BAH	Validation	36–40	Normal	12.06	1.95	10.95	4.82	2.73	2011
62	BAH	Validation	56–60	10, right	10.61	10.85	1382.95	1027.7	0.73	2011
63	BAH	Validation	50–55	Normal	2.5	5.52	52.24	69.78	0.6	2014
64	BAH	Validation	70–75	Normal	2.66	2.27	7.54	10.19	1.58	2011
65	BAH	Validation	50–55	22/11, right/left	5.31	16.92	6.45	7.29	0.35	2013
66	BAH	Validation	50–55	Normal	13.57	8.54	44.95	33.02	1.17	2014
67	BAH	Validation	66–70	5, left	92.8	89.2	696.37	234.54	0.35	2018
68	BAH	Validation	60–65	7, left	35.6	18.22	416.88	199.74	0.94	2016
69	BAH	Validation	36–40	14/20, right/left	83.5	13.2	508.95	181.61	2.26	2018
70	BAH	Validation	66–70	15/10, right/left	4.4	1.7	411.08	235.63	1.48	2018
71	BAH	Validation	66–70	Normal	43.35	21.2	411.44	180.89	0.9	2018
72	BAH	Validation	66–70	7, right	54.5	43.2	317.19	85.19	0.34	2019
73	BAH	Validation	66–70	Normal	99.87	58.26	511.13	291.81	0.98	2018
74	BAH	Validation	36–40	Normal	180.33	21.08	913.51	296.89	2.78	2018
75	BAH	Validation	36–40	5, left	44.39	23.03	114.91	93.16	1.56	2018
76	BAH	Validation	40–45	11, left	11.08	12.07	101.14	128.33	1.16	2019
77	BAH	Validation	50–55	15, right	44.08	15.08	247.73	179.77	2.12	2019
78	APA	Validation	60–65	13, left	125.98	2.12	355.98	437.54	73.05	2018
79	APA	Validation	50–55	20, left	4.82	25.63	330.96	173.28	0.1	2018
80	APA	Validation	20–25	7, left	25.2	47.39	808.02	272.24	0.18	2018
81	APA	Validation	50–55	18, left	7.2	20.8	238.53	61.63	0.09	2018
82	APA	Validation	46–50	23/23, right/left	0.84	47.83	39.51	197.2	0.09	2018
83	APA	Validation	60–65	10, left	5.92	48.19	175.09	69.6	0.05	2018
84	APA	Validation	56–60	10, left	16.9	14.8	379.54	84.46	0.25	2018
85	APA	Validation	56–60	Normal	19.3	41.19	630.03	339.3	0.25	2018
86	APA	Validation	30–35	15, right	18.34	261.18	116.98	70.72	0.04	2018
87	APA	Validation	40–45	20, left	6.63	97.74	191.8	146.89	0.05	2018
88	APA	Validation	56–60	15, right	393.95	17.37	184.51	177.99	21.88	2015
89	APA	Validation	56–60	6/8, right/left	10.26	277.43	193.21	317.91	0.06	2015
90	APA	Validation	40–45	10, right	163.68	3.99	164.94	122.89	30.53	2016
91	APA	Validation	30–35	14, right	108.2	2	97.15	27.55	15.36	2017
92	APA	Validation	46–50	10/15, right/left	1.69	88.78	127.96	124.34	0.02	2017
93	APA	Validation	66–70	12, right	5.55	33.29	192.49	126.51	0.11	2017
94	APA	Validation	56–60	13, left	8.6	244.14	140.65	95.34	0.02	2017
95	APA	Validation	56–60	10/5, right/left	177.56	11.93	314.29	287.1	13.6	2017
96	APA	Validation	50–55	Normal	0.63	6.61	56.91	14.61	0.02	2014
97	APA	Validation	50–55	Normal	50.62	5.93	80.84	266.08	28.11	2014
98	APA	Validation	56–60	16, right	64.6	1.67	174	268.25	59.63	2017
99	APA	Validation	46–50	15, right	160	1.55	12.14	35.71	303.35	2017
100	APA	Validation	36–40	13, left	5.33	62.01	219.68	175.09	0.07	2015
101	APA	Validation	66–70	Normal	15.98	1.13	15.95	104.76	92.96	2014
102	APA	Validation	30–35	13, right	14.32	1.32	28.64	86.28	32.8	2017
103	APA	Validation	50–55	Normal	232.6	0.62	7.98	5.8	273.43	2016
104	APA	Validation	60–65	Normal	25.52	1.31	17.76	63.8	70.16	2014
105	APA	Validation	60–65	20, left	1.12	38.51	12.83	24.14	0.05	2010
106	APA	Validation	56–60	hyperplasia	8.73	7.47	527.44	17.33	0.04	2010
107	APA	Validation	56–60	20/15, right/left	41.76	8.28	572.75	8.59	0.08	2011
108	APA	Validation	36–40	Hyperplasia	1.49	6.33	10.66	9.68	0.21	2013
109	APA	Validation	50–55	40, left	1.35	49.07	78.3	119.63	0.04	2012
110	APA	Validation	56–60	Normal	0.83	97.93	350.18	209.89	0.01	2014
111	APA	Validation	46–50	20, left	1.12	44.72	185.31	210.83	0.03	2015
112	APA	Validation	40–45	Normal	9.73	7.74	10.95	16.97	1.95	2013
113	APA	Validation	36–40	16, right	133.12	0.35	180.13	42.74	91.08	2009
114	APA	Validation	26–30	16, left	3.16	20.91	696.73	441.17	0.1	2011
115	APA	Validation	56–60	12/5, right/left	280.2	10.71	254.11	205.54	21.16	2018
116	APA	Validation	46–50	14, left	8.46	372.42	398.03	379.9	0.02	2018
117	APA	Validation	36–40	6/11, right/left	9.99	99.87	225.48	204.45	0.09	2018
118	APA	Validation	60–65	5/12, right/left	19.7	55.49	265.35	163.49	0.22	2018
119	APA	Validation	50–55	14/13, right/left	61.03	224.72	179.8	84.1	0.13	2018
120	APA	Validation	56–60	9/5, right/left	1417.67	3.83	226.56	82.29	134.49	2019
121	APA	Validation	46–50	18, left	22.97	41.71	89.14	33.42	0.21	2019
122	APA	Validation	66–70	21, right	55.83	1.34	70.76	31.39	18.52	2019
123	APA	Validation	60–65	normal	116.48	19.41	195.21	134.49	4.13	2019

*APA, aldosterone-producing adenoma; AVS, adrenal vein sampling; BAH, bilateral adrenal hyperplasia*.

**Table 2:** List of centers providing samples for the study and AVS strategy.

**Country/Center**	**Sample number**	**AVS protocol**
USA/Rochester	30	ACTH stimulated
Italy/Turin	17	ACTH stimulated
Italy/Padova	13	ACTH stimulated
Croatia/Zagreb	22	ACTH stimulated
Germany/Munich	41	Unstimulated

*ACTH, adrenocorticotropic hormone; AVS, adrenal vein sampling*.

### Sample Processing

Total RNA isolation was carried out from all plasma samples by miRNeasy Serum/Plasma Kit (Qiagen GmbH, Hilden, Germany). For assessing recovery efficacy, 5 μL of 5 nM Syn-cel-miR-39 miScript miRNA Mimic (Qiagen GmbH) was added before the addition of acid-phenol/chloroform as a spike-in control. Total RNA was held frozen at −80°C until further use.

### miRNA Expression Profiling From Plasma Samples by Next-Generation Sequencing (NGS)

A total of 30 samples (16 APA and 14 BAH) were subjected to NGS. APA samples showing the highest (>4) or lowest (<0.25) LI and BAH samples with LI closest to 1 were selected for this cohort. Samples were involved from 3 centers (9, 13, 8 samples, respectively; 22 males and 8 females; average age BAH: 53.28 years, APA: 54.46 years). cDNA library was made from total RNA by the QIAseq miRNA Library Kit (Qiagen GmbH) according to the manufacturer's guideline. The library was prepared for sequencing in accordance with the instructions of the MiSeq Reagent Kit v3 (Illumina, San Diego, CA, USA). NGS was performed by Illumina MiSeq (Illumina). FASTQ files were used in the primary data analysis procedure, in which online analysis software of Qiagen was applied (https://geneglobe.qiagen.com/sg/analyze/). To strengthen our findings, another statistical method was also applied. Primary analysis included trimming of adapters using cutadapt (Marcel Martin, Technical University, Dortmund, Germany); reads with <16 bp insert sequences or with <10 bp Unique Molecular Index were discarded. Alignment of reads was performed using bowtie (John Hopkins University, Baltimore, MD, USA), and miRBase V21 was used for miRNAs. Secondary analysis revealed significantly differently expressed miRNAs after DESeq2 normalization (24). Disease groups were compared by unpaired Mann–Whitney test, and to decrease the false discover rate, corrected *p*-value was calculated by Benjamini–Hochberg method.

### Validation of Individual miRNAs

miRNAs significantly differentially expressed by NGS were validated by RT-qPCR on an independent validation cohort of 93 samples in one center (Semmelweis University, 2nd Department of Internal Medicine). Reverse transcription of RNA was performed using the TaqMan MicroRNA Reverse Transcription Kit (Thermo Fisher Scientific) and individual TaqMan miRNA assays (CN: 4427975, 4440886; Thermo Fisher Scientific). Selected miRNAs were *hsa-miR-30e-5p* (ID: 002223), *hsa-miR-223-3p* (ID: 002295), *hsa-miR-30d-5p* (ID: 000420), and *hsa-miR-7-5p* (ID: 005723_mat). As reference miRNA, *cel-miR-39* (ID: 000200) was used. Quantitative real-time PCR was performed by the TaqMan Fast Universal PCR Master Mix (2x) (CN: 4352042; Thermo Fisher Scientific) on a Quantstudio 7 Flex Real-Time PCR System (Thermo Fisher Scientific) in accordance with the manufacturer's protocol for TaqMan miRNA assays with minor modifications (the end volume of the reaction was 15 μl, program of thermal cycler was the following: after 20 s on 95°C, 40 cycles of 95°C for 3 s and 60°C for 30 s). Negative control reactions contained no cDNA templates. Samples were always run in triplicate. For data evaluation, the dCt method [delta Ct (cycle threshold) value equals target miRNA's Ct minus internal control miRNA's Ct] was used by Microsoft Excel 2016 (Microsoft, Redmond, WA, USA).

### Statistical Analysis

Statistical power analysis was calculated with a statistical power and sample size calculator (HyLown Consulting LLC, Atlanta, GA, USA) (25). RT-qPCR data analysis was performed by GraphPad Prism 7.00 (GraphPad, La Jolla, CA, USA). Being a multicenter study, comparative statistics (Kruskal–Wallis test) were performed on samples from same disease groups, but from different centers in order to find possible skewed results. For differentiating between APA and BAH groups, *t*-test with Welch's correction or Mann–Whitney test based on the result of the Shapiro–Wilk normality test. To exclude skewed results, –dCt values were standardized using standard score (*z*-value, *z*-score: $z=\frac{x-\mu }{\sigma }$, where μ and σ is the mean and standard deviation of values of the given center, respectively. The *F*-test was used to evaluate differences between variances of circulating miRNA expressions of APA and BAH. Receiver operating characteristic (ROC) analysis was performed on miRNAs that could have potential utility as minimally invasive biomarkers. *P* < 0.05 were considered significant.

## Results

### miRNA Expression Profiling by NGS

We found 50 miRNAs to be significantly differentially expressed in samples of patients with APA vs. samples of patients with BAH by NGS and analyzed with the Qiagen online software. Multiple statistical analysis (including unpaired Mann–Whitney test) was performed on primary data that resulted in nine miRNAs showing the highest levels of significance (Table 3). From these, four miRNAs with the highest significance i.e., *hsa-miR-30e-5p* (*p*-value: 0.0005), *hsa-miR-223-3p* (*p*-value: 0.0039)*, hsa-miR-30d-5p* (*p*-value: 0.0091), and *hsa-miR-7-5p* (*p*-value: 0.0134) were selected for validation on an independent cohort of samples. Statistical power analysis showed that by using this cohort of samples, the power of the sequencing was above 0.99. NGS data are available under the Gene Expression Omnibus (GEO) accession number GSE126386.

**Table 3:** List of 9 miRNAs with the most significant differences in expression between APA and BAH samples.

**miRNA**	***p*-value**
**hsa-miR-30e-5p**	**0.000506**
**hsa-miR-223-3p**	**0.003865**
**hsa-miR-30d-5p**	**0.009064**
**hsa-miR-7-5p**	**0.013403**
hsa-let-7d-3p	0.024542
hsa-miR-16-5p	0.038273
hsa-miR-19b-3p	0.042548
hsa-miR-339-3p	0.042548
hsa-miR-22-3p	0.047205

*In bold, the four microRNAs with the highest level of significance that have been subjected to validation*.

### Real-Time qPCR Validation of Selected miRNAs

**Figure 1:**
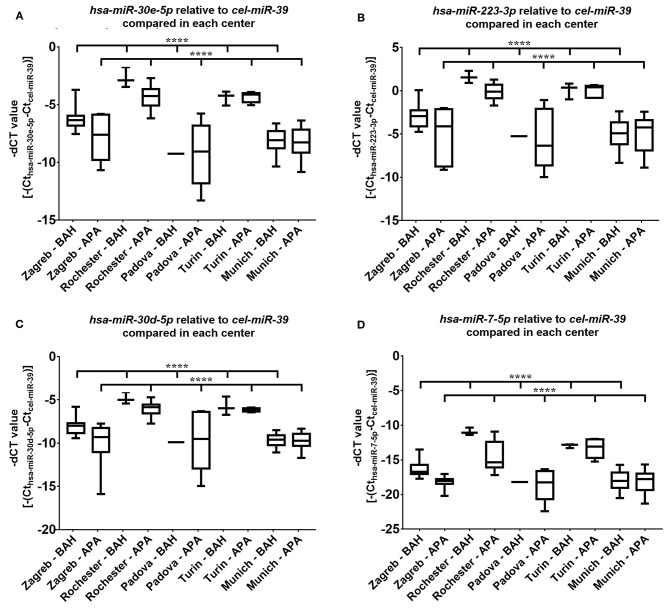
Results of RT-qPCR of the four miRNAs selected for validation from each sample contributing center. Mean ± Standard deviation (SD) of -dCT values of selected miRNAs; **(A)** *hsa-miR-30e-5p*; **(B)** *hsa-miR-223-3p*; **(C)** *hsa-miR-30d-5p*; **(D)** *hsa-miR-7-5p*. Significant differences can be seen among the APA or BAH samples from different centers (ANOVA or Kruskal–Wallis test based on the result of Shapiro–Wilks normality test). ^****^p < 0.0001.

**Figure 2:**
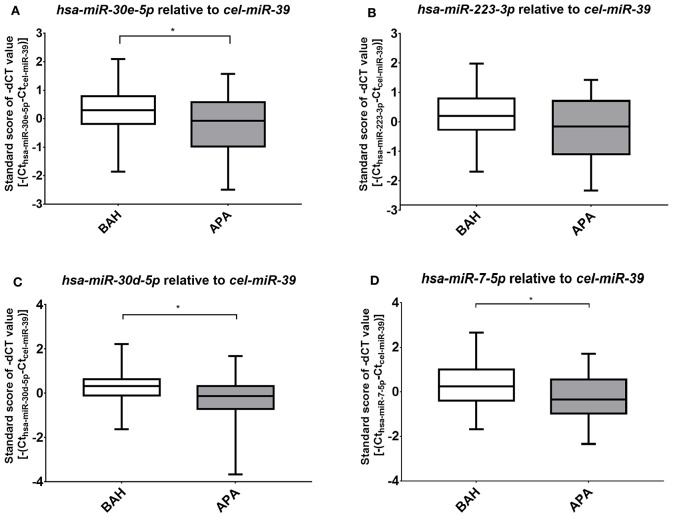
Results of RT-qPCR of the four miRNAs selected for validation. Mean ± Standard deviation (SD) of standard scores of -dCT values of selected miRNAs; **(A)** *hsa-miR-30e-5p*; **(B)** *hsa-miR-223-3p*; **(C)** *hsa-miR-30d-5p*; **(D)** *hsa-miR-7-5p*. Student's *t*-test with Welch correction or Mann–Whitney test was used based on the result of the Shapiro–Wilks normality test. ^*^p < 0.05.

Four miRNAs, *hsa-miR-7-5p, hsa-miR-30d-5p, hsa-mir-30e-5p*, and *hsa-miR-223-3p* were subjected to validation by real-time RT-qPCR on 93 samples. Differences between miRNA expression within the investigated disease groups (APA and BAH) between different centers could be demonstrated (*p* < 0.0001), but the higher expression of miRNA in BAH relative to APA is evident for most cases (Figure 1). To exclude distorted results, standard scores of miRNA expression values of APA and BAH samples were compared (Figure 2). Validation of three out of four miRNAs established as significant by NGS were successful. *Hsa-miR-30e-5p* (*p* = 0.04) (Figure 2A), *hsa-miR-30d-5p* (*p* = 0.02) (Figure 2C), and *hsa-miR-7-5p* (*p* = 0.016) (Figure 2D) were significantly upregulated in BAH in comparison with APA samples. An upregulation tendency of *hsa-miR-223-3p* in BAH samples relative to APA samples was noticeable, but not significant (*p* = 0.15) (Figure 2B). As shown on Figure 1 regarding the relative differences between standard deviations, BAH samples appear to be homogenous at the level of miRNA expression, while miRNA expression in APA samples are more heterogeneous. To evaluate difference between variances of sample groups we applied *F*-test. *P*-values for *hsa-miR-7-5p* were: Zagreb: 0.35; Rochester: 0.055; Padova: n.d.; Turin: 0.03; Munich: 0.24, if *p* < 0.05, null-hypothesis is rejected, therefore standard deviations are surely not equal. Relative miRNA expression did not correlate with any of the measured parameters (tumor diameter, lateralization index, aldosterone ratio between two sides at AVS, basal peripheral aldosterone) and no sex difference was observed.

### Diagnostic Performance of Circulating miRNAs

**Figure 3:**
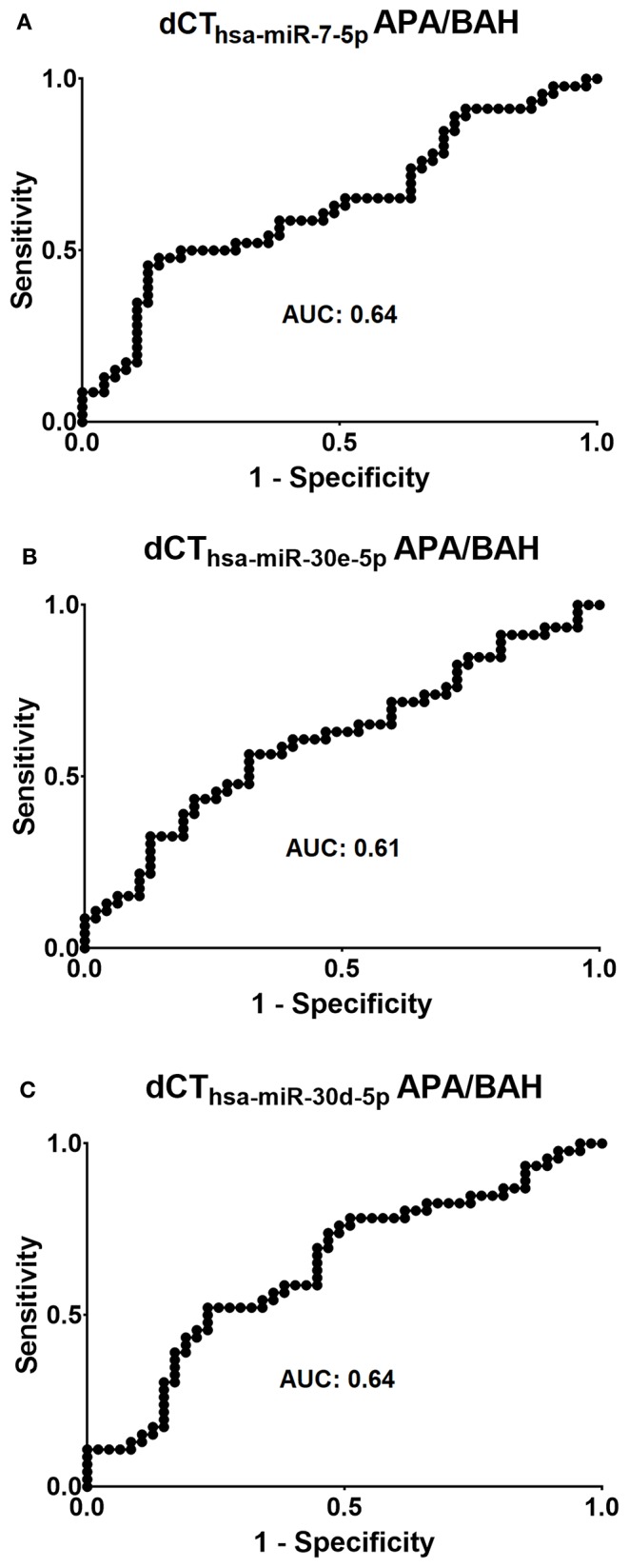
Evaluation of the diagnostic applicability of **(A)** *hsa-miR-7-5p*, **(B)** *hsa-miR-30e-5p*, and **(C)** *hsa-miR-30d-5p* by receiver operating characteristic (ROC) curves. AUC = area under curve.

The diagnostic utility of the three significantly differentially expressed circulating microRNAs, *hsa-miR-7-5p, hsa-miR-30e-5p*, and *hsa-miR-30d-5p* was evaluated by ROC analysis (Figure 3). For *hsa-miR-7-5p*, the area under curve (AUC) was 0.64, and specificity and sensitivity values were 61.7 and 58.7%, respectively when choosing 0.13 as a cut-off point. ROC-analysis of *hsa-miR-30e-5p* showed an AUC of 0.61, and sensitivity of 58.7% and specificity of 61.7% when choosing 0.06 as a cut-off point. *Hsa-miR-30d-5p* performed similarly to *hsa-miR-7-5p* on ROC-analysis, AUC: 0.64, sensitivity: 58.7% and specificity: 61.7% when choosing a cut-off point of 0.05.

## Discussion

Several genes have been described to be involved in the pathogenesis of APA, but the pathogenesis of BAH remains elusive. A recent study reported that aldosterone-producing cell clusters can be detected in BAH, and in these *CACNA1D* and *KCNJ5* mutations were found (26). BAH could be associated with bilateral microscopic hyperplasia, bilateral nodular hyperplasia, bilateral adenomas, or bilateral adrenal aldosterone-producing cell clusters (27). Based on recent data, the various forms of PA can be regarded as representatives of a spectrum of diseases of variable severity (28, 29).

From a clinical perspective, differentiation of a unilateral APA from a bilateral hyperplasia is of pivotal importance, as their treatment is different (operation vs. medical therapy). AVS is the gold standard, but it is not widely available, it is invasive and requires great expertise. A minimally invasive marker for differentiating these two entities would be an invaluable help in the management of PA. We have therefore examined the expression of circulating miRNA in AVS-confirmed APA and BAH samples to evaluate the applicability of these novel epigenetic markers for their differentiation.

In our study, 50 miRNAs showed some degree of significantly different expression by NGS, and from the four miRNA selected for validation, three circulating miRNAs *hsa-miR-30e-5p, hsa-miR-30-5p*, and *hsa-miR-7-5p* were confirmed to be significantly up-regulated in BAH in comparison with APA (the fourth studied miRNA *hsa-miR-223-3p* showed only a non-significant tendency of up-regulation in BAH). In a previous study of miRNA expression in PA, where the authors compared tissue miRNA expression profiles of APA, unilateral adrenal hyperplasia (UAH) and normal adrenal cortex, *hsa-miR-375* and *hsa-miR-7* were significantly underexpressed in APA when compared to UAH and normal adrenal glands (30). Moreover, in a recent study, three of our selected circulating miRNAs *hsa-miR-30e-5p, hsa-miR-30d-5p*, and *hsa-miR-223-3p* were found to be down-regulated in essential hypertension patients compared to healthy people's plasma samples (31). These observations could raise the possibility that these miRNAs might be related to the regulation of blood pressure.

The range of expression of all four validated miRNAs seems to be broader in APA samples than in BAH samples (*F*-test was significantly different for data of two centers, and a tendency was seen in another). This finding might be related to the observations, that APA is genetically more heterogeneous than BAH (10, 11).

It is unclear why the expression levels (represented by dCt values) in the APA and BAH groups from different centers contributing to our study are different. The tendency of up-regulation of miRNA in BAH relative to APA can be seen for most miRNAs, however, the expression levels were rather different between some centers (Figure 1). Pre-analytical differences such as sample taking/storage might be suspected.

Despite showing significant overexpression in BAH samples, the diagnostic accuracy of the three validated circulating miRNAs (*hsa-miR-30e-5p, hsa-miR-30-5p*, and *hsa-miR-7-5p*) does not make them suitable for introduction to clinical practice. In contrast, adrenal venous sampling has impressive sensitivity and specificity values–when lateralization index cut-off point is 4–with 95.2 and 100%, respectively (23).

The pathogenic relevance of these miRNA in PA is unclear. Circulating *hsa-miR-7-5p* is found to be underexpressed in idiopathic inflammatory myopathy and esophageal squamous cell cancer patients compared to healthy controls (32, 33). Overexpressed *hsa-miR-7-5p* was found in acute pancreatitis, neuroendocrine tumors, and type 2 diabetes mellitus patients compared to healthy controls (34–36). There are reports stating that *hsa-miR-7-5p* functions as a tumor suppressor miRNA in pancreatic ductal adenocarcinoma (37) and in bladder cancer (38), and also inhibits melanoma cell proliferation (39). Circulating *hsa-miR-30e-5p* is up-regulated in systemic lupus erythematosus patients (40) and down-regulated in patients with mitral chord rupture (41) compared to healthy controls. Tissue *hsa-miR-30d-5p* is considered as a tumor suppressor miRNA in non-small cell lung cancer compared to healthy controls (42).

There are limitations of our study. Even if adrenal imaging was performed for all patients, due to the limited sensitivity of computed tomography and magnetic resonance imaging, bilateral adrenal microadenomas can be classified as bilateral hyperplasia. Actually, as the group of William E. Rainey has recently shown (26), BAH usually contains microadenomas, and thus the boundary between APA and BAH is not clear, and these PA forms can be regarded as representatives of the same spectrum of diseases. The clinical relevance, however, is still to be able to differentiate unilateral from bilateral forms. It would also be interesting to assess the circulating miRNA expression profiles related to different genetic forms of APA, but this would exceed the scope of our present study where the comparison of unilateral with bilateral forms of APA has been the primary aim for evaluating the potential applicability of circulating miRNA as markers of lateralization. Heterogeneity among contributing centers is another limitation, as discussed above.

To summarize, we have found that three circulating microRNAs were significantly overexpressed in BAH compared to APA patients, but don't have high enough sensitivity and specificity values to be introduced to clinical medicine. BAH seems to be more homogeneous in miRNA expression than APA. These findings also seem to support the idea that APA and BAH represent entities forming part of a spectrum of diseases leading to primary aldosteronism.

## Data Availability Statement

The datasets generated for this study can be found under the Gene Expression Omnibus (GEO) accession number GSE126386. The datasets generated during PCR validation are not publicly available, but are available from the corresponding author on reasonable request.

## Ethics Statement

The studies involving human participants were reviewed and approved by Ethical Committee of the Hungarian Health Council. The patients/participants provided their written informed consent to participate in this study.

## Author Contributions

PI designed the research. AD, GN, and PT performed the research. IB, RK, RP, MI, IK, DK, MP-C, MM, NN, DH, and MR provided patient samples. OD and AP were involved in data analysis. AD and PI wrote the manuscript. All authors approved the final manuscript.

### Conflict of Interest

The authors declare that the research was conducted in the absence of any commercial or financial relationships that could be construed as a potential conflict of interest.

## References

[1] Rossi GP Bernini G Caliumi C Desideri G Fabris B Ferri C. A prospective study of the prevalence of primary aldosteronism in 1,125 hypertensive patients. J Am Coll Cardiol. (2006) 48:2293–300. 10.1016/j.jacc.2006.07.059 17161262

[2] Loh K-C Koay ES Khaw M-C Emmanuel SC Young WF Prevalence of primary aldosteronism among Asian hypertensive patients in Singapore 1. J Clin Endocrinol Metab. (2000) 85:2854–9. 10.1210/jcem.85.8.6752 10946893

[3] Käyser SC Dekkers T Groenewoud HJ Van Der Wilt GJ Carel Bakx J Van Der Wel MC. Study heterogeneity and estimation of prevalence of primary aldosteronism: a systematic review and meta-regression analysis. J Clin Endocrinol Metab. (2016) 101:2826–35. 10.1210/jc.2016-1472 27172433

[4] Young WF. Primary aldosteronism: renaissance of a syndrome. Clin Endocrinol. (2007) 66:607–18. 10.1111/j.1365-2265.2007.02775.x 17492946

[5] Boulkroun S Beuschlein F Rossi GP Golib-Dzib JF Fischer E Amar L. Prevalence, clinical, and molecular correlates of KCNJ5 mutations in primary aldosteronism. Hypertension. (2012) 59:592–8. 10.1161/HYPERTENSIONAHA.111.186478 22275527

[6] Azizan EAB Murthy M Stowasser M Gordon R Kowalski B Xu S. Somatic mutations affecting the selectivity filter of KCNJ5 are frequent in 2 large unselected collections of adrenal aldosteronomas. Hypertension. (2012) 59:587–91. 10.1161/HYPERTENSIONAHA.111.186239 22252394

[7] Monticone S Hattangady NG Nishimoto K Mantero F Rubin B Cicala MV. Effect of KCNJ5 mutations on gene expression in aldosterone-producing adenomas and adrenocortical cells. J Clin Endocrinol Metab. (2012) 97:1567–72. 10.1210/jc.2011-3132 22628608 PMC3410264

[8] Åkerström T Crona J Delgado Verdugo A Starker LF Cupisti K Willenberg HS. Comprehensive re-sequencing of adrenal aldosterone producing lesions reveal three somatic mutations near the KCNJ5 potassium channel selectivity filter. PLoS ONE. (2012) 7:41926. 10.1371/journal.pone.0041926 22848660 PMC3407065

[9] Williams TA Monticone S Mulatero P. *KCNJ5* mutations are the most frequent genetic alteration in primary aldosteronism. Hypertension. (2015) 65:507–9. 10.1161/HYPERTENSIONAHA.114.04636 25624337

[10] Beuschlein F Boulkroun S Osswald A Wieland T Nielsen HN Lichtenauer UD. Somatic mutations in ATP1A1 and ATP2B3 lead to aldosterone-producing adenomas and secondary hypertension. Nat Genet. (2013) 45:440–4. 10.1038/ng.2550 23416519

[11] El Zein RM Boulkroun S Fernandes-Rosa FL Zennaro M-CC. Molecular genetics of Conn adenomas in the era of exome analysis. Press Med. (2018) 47:151–8. 10.1016/j.lpm.2018.07.006 30072045

[12] Fernandes-Rosa FL Boulkroun S Zennaro M-C. Somatic and inherited mutations in primary aldosteronism. J Mol Endocrinol. (2017) 59:R47–63. 10.1530/JME-17-0035 28400483

[13] Nanba K Omata K Else T Beck PCC Nanba AT Turcu AF. Targeted molecular characterization of aldosterone-producing adenomas in white Americans. J Clin Endocrinol Metab. (2018) 103:3869–76. 10.1210/jc.2018-01004 30085035 PMC6179168

[14] Fernandes-Rosa FL Williams TA Riester A Steichen O Beuschlein F Boulkroun S. Genetic spectrum and clinical correlates of somatic mutations in aldosterone-producing adenoma. Hypertension. (2014) 64:354–61. 10.1161/HYPERTENSIONAHA.114.03419 24866132

[15] Rossi GP Barisa M Allolio B Auchus RJ Amar L Cohen D. The Adrenal Vein Sampling International Study (AVIS) for identifying the major subtypes of primary aldosteronism. J Clin Endocrinol Metab. (2012) 97:1606–14. 10.1210/jc.2011-2830 22399502

[16] Rossi GP Auchus RJ Brown M Lenders JWM Naruse M Plouin PF. An expert consensus statement on use of adrenal vein sampling for the subtyping of primary aldosteronism. Hypertension. (2014) 63:151–60. 10.1161/HYPERTENSIONAHA.113.02097 24218436

[17] Lenders J Eisenhofer G Reincke M. Subtyping of patients with primary aldosteronism: an update. Horm Metab Res. (2017) 49:922–8. 10.1055/s-0043-122602 29202492

[18] Beuschlein F Mulatero P Asbach E Monticone S Catena C Sechi L. The SPARTACUS trial: controversies and unresolved issues. Horm Metab Res. (2017) 49:936–42. 10.1055/s-0043-120524 29165736

[19] Bartel DP. MicroRNAs: genomics, biogenesis, mechanism, and function. Cell. (2004) 116:281–97. 10.1016/S0092-8674(04)00045-5 14744438

[20] Malumbres M. miRNAs and cancer: an epigenetics view. Mol Aspects Med. (2013) 34:863–74. 10.1016/j.mam.2012.06.005 22771542 PMC5791883

[21] Weber JA Baxter DH Zhang S Huang DY Huang KH Lee MJ. The microRNA spectrum in 12 body fluids. Clin Chem. (2010) 56:1733–41. 10.1373/clinchem.2010.147405 20847327 PMC4846276

[22] Funder JW Carey RM Mantero F Murad MH Reincke M Shibata H. The management of primary aldosteronism: case detection, diagnosis, and treatment: an endocrine society clinical practice guideline. J Clin Endocrinol Metab. (2016) 101:1889–916. 10.1210/jc.2015-4061 26934393

[23] Young WF Stanson AW Thompson GB Grant CS Farley DR van Heerden JA. Role for adrenal venous sampling in primary aldosteronism. Surgery. (2004) 136:1227–35. 10.1016/j.surg.2004.06.051 15657580

[24] Love MI Huber W Anders S. Moderated estimation of fold change and dispersion for RNA-seq data with DESeq2. Genome Biol. (2014) 15:550. 10.1186/s13059-014-0550-8 25516281 PMC4302049

[25] Chow S-C Shao J Wang H Comparing means. In: Chow SC editor. Sample Size Calculations in Clinical Research. Boca Raton, FL: Chapman & Hall/CRC (2008). p. 451.

[26] Omata K Satoh F Morimoto R Ito S Yamazaki Y Nakamura Y. Cellular and genetic causes of idiopathic hyperaldosteronism. Hypertension. (2018) 72:874–80. 10.1161/HYPERTENSIONAHA.118.11086 30354720 PMC6207209

[27] Scholl U. Unanswered questions in the genetic basis of primary aldosteronism. Horm Metab Res. (2017) 49:963–8. 10.1055/s-0043-120066 29065434

[28] Gomez-Sanchez CE Rossi GP Fallo F Mannelli M. Progress in primary aldosteronism: present challenges and perspectives. Horm Metab Res. (2010) 42:374–81. 10.1055/s-0029-1243619 20091458 PMC4823770

[29] Derwahl M Studer H. Hyperplasia versus adenoma in endocrine tissues: are they different? Trends Endocrinol Metab. (2002) 13:23–8. 10.1016/S1043-2760(01)00519-7 11750859

[30] He J Cao Y Su T Jiang Y Jiang L Zhou W. Downregulation of miR-375 in aldosterone-producing adenomas promotes tumour cell growth via MTDH. Clin Endocrinol (Oxf). (2015) 83:581–9. 10.1111/cen.12814 25944465

[31] Ye Y Yang J Lv W Lu Y Zhang L Zhang Y. Screening of differentially expressed microRNAs of essential hypertension in Uyghur population. Lipids Health Dis. (2019) 18:98. 10.1186/s12944-019-1028-1 30975221 PMC6460779

[32] Yu L Li J Chen Y Jiang J Fang Q Jiang J. hsa-miR-7 is a potential biomarker for idiopathic inflammatory myopathies with interstitial lung disease in humans. Ann Clin Lab Sci. (2018) 48:764–9. 30610047

[33] Dong W Li B Wang J Song Y Zhang Z Fu C. Diagnostic and predictive significance of serum microRNA-7 in esophageal squamous cell carcinoma. Oncol Rep. (2016) 35:1449–56. 10.3892/or.2015.4499 26708917

[34] Lu P Wang F Wu J Wang C Yan J Li Z-L. Elevated serum miR-7, miR-9, miR-122, and miR-141 are noninvasive biomarkers of acute pancreatitis. Dis Mark. (2017) 2017:7293459. 10.1155/2017/7293459 29332987 PMC5733206

[35] Wan S Wang J Wang J Wu J Song J Zhang C-Y. Increased serum miR-7 is a promising biomarker for type 2 diabetes mellitus and its microvascular complications. Diabetes Res Clin Pract. (2017) 130:171–9. 10.1016/j.diabres.2017.06.005 28646700

[36] Heverhagen AE Legrand N Wagner V Fendrich V Bartsch DK Slater EP. Overexpression of microRNA miR-7-5p is a potential biomarker in neuroendocrine neoplasms of the small intestine. Neuroendocrinology. (2018) 106:312–7. 10.1159/000480121 28848144

[37] Zhu W Wang Y Zhang D Yu X Leng X. MiR-7-5p functions as a tumor suppressor by targeting SOX18 in pancreatic ductal adenocarcinoma. Biochem Biophys Res Commun. (2018) 497:963–70. 10.1016/j.bbrc.2018.02.005 29408481

[38] Li J Qiu M An Y Huang J Gong C. miR-7-5p acts as a tumor suppressor in bladder cancer by regulating the hedgehog pathway factor Gli3. Biochem Biophys Res Commun. (2018) 503:2101–7. 10.1016/j.bbrc.2018.07.166 30100065

[39] Giles KM Brown RAM Ganda C Podgorny MJ Candy PA Wintle LC. microRNA-7-5p inhibits melanoma cell proliferation and metastasis by suppressing RelA/NF-κB. Oncotarget. (2016) 7:31663–80. 10.18632/oncotarget.9421 27203220 PMC5077967

[40] Kim B-S Jung J-Y Jeon J-Y Kim H-A Suh C-H. Circulating hsa-miR-30e-5p, hsa-miR-92a-3p, and hsa-miR-223-3p may be novel biomarkers in systemic lupus erythematosus. HLA. (2016) 88:187–93. 10.1111/tan.12874 27596248

[41] Bulent Vatan M Kalayci Yigin A Akdemir R Tarik Agac M Akif Cakar M Aksoy M. Altered plasma microRNA expression in patients with mitral chordae tendineae rupture. J Heart Valve Dis. (2016) 25:580–8. 28238240

[42] Hosseini SM Soltani BM Tavallaei M Mowla SJ Tafsiri E Bagheri A. Clinically significant dysregulation of hsa-miR-30d-5p and hsa-let-7b expression in patients with surgically resected non-small cell lung cancer. Avicenna J Med Biotechnol. (2018) 10:98–104. 29849986 PMC5960066

